# Deciphering a cell death-associated signature for predicting prognosis and response to immunotherapy in lung squamous cell carcinoma

**DOI:** 10.1186/s12931-023-02402-9

**Published:** 2023-07-06

**Authors:** Guangxian Mao, Dongyong Yang, Bin Liu, Yu Zhang, Sijia Ma, Shang Dai, Guoqiang Wang, Wenxiang Tang, Huafei Lu, Shangli Cai, Jialiang Zhu, Huaping Yang

**Affiliations:** 1 Department of Thoracic Surgery, Peking University Shenzhen Hospital, Shenzhen, 518036 China; 2 Department of Pulmonary and Critical Care Medicine, Respiratory Medicine Center of Fujian Province, Second Affiliated Hospital of Fujian Medical University, Guangzhou, 362000 China; 3 First Division, Department of Respiratory and Critical Care Medicine, Affiliated to Xiangya School of Medicine, Zhuzhou Hospital, Central South University, Zhuzhou Central Hospital, Zhuzhou, 412007 China; 4 Burning Rock Biotech, Guangzhou, 510300 China; 5 Department of General Practice, the Third Xiangya Hospital of Central South University, Changsha, 410013 China; 6 Department of Cardiothoracic Surgery, the Third Xiangya Hospital of Central South University, 138 Tongzipo Road, Yuelu District, Changsha, 410013 China; 7 Department of Respiratory Medicine, Xiangya Hospital, Central South University, 87 Xiangya Road, Changsha, 410008 China

**Keywords:** Lung squamous cell carcinoma, Cell death, Prognostic signature, Immunotherapeutic response, Cytokines

## Abstract

**Background:**

Lung squamous cell carcinoma (LUSC) is a subtype of non-small cell carcinoma, accounting for about 30% of all lung cancers. Yet, the evaluation of prognostic outcome and therapy response of patients with LUSC remains to be resolved. This study aimed to explore the prognostic value of cell death pathways and develop a cell death-associated signature for predicting prognosis and guiding treatment in LUSC.

**Methods:**

Transcriptome profiles and corresponding clinical information of LUSC patients were gathered from The Cancer Genome Atlas (TCGA-LUSC, n = 493) and Gene Expression Omnibus database (GSE74777, n = 107). The cell death-related genes including autophagy (n = 348), apoptosis (n = 163), and necrosis (n = 166) were retrieved from the Kyoto Encyclopedia of Genes and Genomes and Gene Ontology databases. In the training cohort (TCGA-LUSC), LASSO Cox regression was used to construct four prognostic signatures of respective autophagy, apoptosis, and necrosis pathway and genes of three pathways. After comparing the four signatures, the cell death index (CDI), the signature of combined genes, was further validated in the GSE74777 dataset. We also investigated the clinical significance of the CDI signature in predicting the immunotherapeutic response of LUSC patients.

**Results:**

The CDI signature was significantly associated with the overall survival of LUSC patients in the training cohort (HR, 2.13; 95% CI, 1.62‒2.82; *P* < 0.001) and in the validation cohort (HR, 1.94; 95% CI, 1.01‒3.72; *P* = 0.04). The differentially expressed genes between the high- and low-risk groups contained cell death-associated cytokines and were enriched in immune-associated pathways. We also found a higher infiltration of naive CD4^+^ T cells, monocytes, activated dendritic cells, neutrophils, and lower infiltration of plasma cells and resting memory CD4^+^ T cells in the high-risk group. Tumor stemness indices, mRNAsi and mDNAsi, were both negatively correlated with the risk score of the CDI. Moreover, LUSC patients in the low-risk group are more likely to respond to immunotherapy than those in the high-risk group (*P* = 0.002).

**Conclusions:**

This study revealed a reliable cell death-associated signature (CDI) that closely correlated with prognosis and the tumor microenvironment in LUSC, which may assist in predicting the prognosis and response to immunotherapy for patients with LUSC.

**Supplementary Information:**

The online version contains supplementary material available at 10.1186/s12931-023-02402-9.

## Background

Lung cancer accounted for 11.4% and 18% of the total cancer incidence and mortality worldwide, respectively [1]. In the United States, 236,740 new cases and 130,180 deaths of lung cancer were estimated to occur in 2022 [2]. As a common pathological type of non-small cell lung cancer (NSCLC), lung squamous cell carcinoma (LUSC) comprises about 30% of all cases [3]. The prognosis and treatment decision of LUSC patients is mostly evaluated based on the American Joint Committee on Cancer (AJCC) staging system (eighth edition). Yet, this system may not able to distinguish the heterogeneous outcomes of individual patients with the same TNM stage because of other complicated factors [4, 5]. Thus, novel biomarkers and prognostic signatures for identifying LUSC patients at higher risk are required to optimize clinical management.

Cell death and related mechanisms are pivotal during the evolution of diseases. Apoptosis, autophagy, and necrosis are the three main types of mammalian cell death [6], which have been widely studied in tumorigenesis. Apoptosis mediates cell death through three distinct pathways, i.e., death receptors, mitochondria, and endoplasmic reticulum, along with the activation of effector cysteinyl aspartate proteases (caspases) [7]. Eluding apoptosis is an important hallmark of cancer. Autophagy is a cellular catabolic degradation in response to starvation or stress whereby macromolecules, organelles, and cytoplasm are engulfed, digested, and recycled to sustain cellular metabolism to generate energy and metabolites [8]. Autophagy can also eliminate pathogens, superfluous cytoplasmic components, and damaged or apoptotic cells [9, 10]. Although presumably as a suppressor of neoplasia, autophagy dysregulation may be a key mechanism in tumor progression to enable long-term survival, tolerance from cancer therapy, regrowth, and eventual relapse [11]. Necrosis has long been considered as a result of a response to acute hypoxic or ischemic injury, occurring spontaneously in neoplasms when cell proliferation outpaces angiogenesis [6, 12].

Cell death plays a key role in various immunological processes associated with tumor progression and drug resistance. Immune cells in the tumor microenvironment (TME) function as dual roles in both tumor-antagonizing and tumor-promoting, and immunogenic cell death occupies features prominently in stimulating the dysfunctional antitumor immune system [13, 14]. Recently, immunotherapy has developed rapidly in lung cancer. The immune checkpoint inhibitors (ICIs) effectively improve the survival of patients with advanced LUSC by targeting programmed cell death protein 1 (PD-1) and its ligand PD-L1, such as pembrolizumab [15], nivolumab [16], and atezolizumab [17]. Immunotherapy combined with classic chemotherapy or radiotherapy and targeted therapies can induce apoptosis of cancer cells through granzyme or extrinsic cell death [18]. Certain macrophages or dendritic cells that engulf apoptotic cells can subsequently activate antitumor immunity [19]. Besides, a subset of caspases has been identified to be involved in immune responses to pathogens and associated with the maturation of pro-inflammatory cytokines [20]. Given the rapid progress of immunotherapy, more detailed findings of cell death might create a new dimension to develop accurate risk stratification, prognostic evaluation model, and personalized immunotherapy for patients with cancer.

Bioinformatic analysis of genomic, transcriptomic, or proteomic profiles has identified a number of potential biomarkers for LUSC [21–24]. However, little research on cell death-related signatures in LUSC, which has been established in lung adenocarcinoma (LUAD) [25, 26] and other types of cancer [27, 28]. In this systematic study, a robust prognostic signature based on cell death-related genes was constructed based on the cell death-associated genes and further confirmed as an independent prognostic factor for LUSC. Most importantly, we highlighted the crucial role of this signature in predicting the survival and immunotherapeutic response of patients with LUSC.

## Methods

### Data acquisition and preprocessing

Transcriptome profiles and clinical data of two public cohorts of LUSC patients were respectively collected from the databases of The Cancer Genome Atlas (TCGA) and Gene Expression Omnibus (GEO). Patients without complete clinical and survival information were excluded from the study (11 cases in the TCGA-LUSC cohort). The RNA-Seq data of 493 LUSC tissue and 49 adjacent normal tissue samples in the training cohort were from the TCGA-LUSC dataset retrieved from UCSC Xena (https://gdc.xenahubs.net) and converted into transcript per million (TPM) formats before analysis. For the GSE74777 validation cohort (n = 107), the raw CEL files generated by Affymetrix were downloaded from the GEO dataset (http://www.ncbi.nlm.nih.gov/geo/). The probes in each dataset were annotated according to the platform annotation file. When multiple probes were mapped to the same gene, the average expression value of the gene was used as the final value. The baseline characteristics of LUSC samples are provided in Table S1.

To determine the prognostic value of cell death-related genes in LUSC, a list of genes in the pathways of autophagy (n = 348), apoptosis (n = 163), and necrosis (n = 166) were gathered from the Kyoto Encyclopedia of Genes and Genomes (KEGG) and Gene Ontology (GO) databases (https://www.gsea-msigdb.org) using terms “KEGG_Regulation of autophagy”, “GOBP_Execution phase of apoptosis”, “KEGG_Apoptosis”, and “GOBP_Tumor necrosis factor mediated signaling pathway”. A total of 263 cytokine genes were downloaded using the keyword “KEGG_Cytokine-cytokine receptor interaction” (https://www.gsea-msigdb.org). These genes are listed in Table S2.

### Construction and validation of cell death-related prognostic signature

In the training cohort, TCGA-LUSC, univariable Cox analysis was utilized to screen the cell death-related genes with prognostic value. Then, the least absolute shrinkage and selection operator (LASSO) logistic regression algorithm was used to reduce the candidate genes and minimize the risk of overfitting. Univariable cox regression to screen genes associated with three cell death pathways of autophagy, apoptosis, and necrosis was conducted by R package “glmnet”. Then, the screened genes were included in the construction of four risk-scoring models by LASSO Cox regression. The penalty parameter (λ) that was chosen as the minimal value of partial likelihood deviance in the LASSO, of each prognostic signature was determined by 10-fold cross-validation.

The risk scores of samples for each signature were calculated according to the expression level of each cell death-related gene and its corresponding regression coefficient. The formula was established as follows:

$${\rm{Risk}}\_scores = \sum {Coef(i)*Exp(i)}$$

According to the median risk score, samples were divided into high- and low-risk groups. Kaplan-Meier survival analysis was implemented to compare the survival between the two groups. A time-dependent receiver operating characteristic (ROC) curve was conducted to evaluate the predictive value of the prognostic signature. Multivariable Cox analysis was performed to verify the independent prognostic value of the cell death-related prognostic model.

The concordance index (C-index) was calculated to evaluate the performance of the signature by R package “survival” and the value ranged from 0.5 to 1.0. The value of 0.5 and 1.0 respectively represented random opportunities and excellent feasibility of a prognostic signature in predicting the survival of LUSC patients.

### Differential expression and functional enrichment analysis

The differentially expressed genes (DEGs) between high- and low-risk groups were identified using the “limma” package in R, which was used to conduct difference analyses by estimating the mean and variance of gene expression in different groups through linear models. The filtering threshold was set as false discovery rate (FDR) < 0.01 and absolute log_2_fold change > 0.4. GO annotation, and KEGG analysis was carried out using the Database for Annotation, Visualization, and Integrated Discovery (DAVID). The FDR was used for the *P* value correction.

### Protein–protein interaction (PPI) network analysis

The interaction associations of the proteins were analyzed using the online tool Search Tool for the Retrieval of Interacting Genes (STRING; http://string-db.org/) and visualized by Cytoscape software. A confidence level (combined score) ≥ 0.7 was selected as the cutoff criterion.

### Immune cell infiltration analysis

The Cell Type Identification by Estimating Relative Subsets of RNA Transcripts (CIBERSORT) deconvolution algorithm (https://cibersortx.stanford.edu/) is an analytical tool reinforced by support vector regression to quantify the abundances of each cell type in a mixed cell population using gene expression profiles. The abundance of 22 types of infiltrating immune cells of each patient with LUSC in the TCGA cohort was estimated by translating the gene expression level into the relative proportion of immune cells using CIBERSORT.

### Immunotherapeutic response prediction

The Tumor Immune Dysfunction and Exclusion (TIDE) algorithm (http://tide.dfci.harvard.edu/) and unsupervised subclass mapping analysis (GenePattern module “SubMap”; https://cloud.genepattern.org/gp/) were performed to predict the treatment response of LUSC patients with high- or low-risk scores to immunotherapy. TIDE is a computational method that integrates the expression profile of T cell dysfunction and exclusion to model tumor immune escape. Submap is an algorithm to assess similarities in gene expression between previously defined immunophenotypes and responders or non-responders to ICIs.

### Statistical analysis

All statistical analyses were performed using the R statistical software, version 3.5.3 (https://www.r-project.org). ROC analysis was performed to describe the discrimination accuracy of cell death-related prognostic signatures for patients with LUSC. The area under the curve (AUC) represented the accuracy and efficiency of this signature as a predictive tool for prognosis. Kaplan-Meier curves with log-rank tests were used to compare the differences in survival between the two groups. Univariable and multivariable Cox regression analyses were utilized to screen the independent predictors for the survival of LUSC patients. The Wilcoxon rank sum test was used to compare the different abundance of 22 immune cells between high- and low-risk groups. Spearman correlation was employed to test the correlation between risk score and tumor stemness indices. *P* value < 0.05 was considered statistically significant.

## Results

### Construction of cell death-associated prognostic signatures for lung squamous cell carcinoma

**Fig. 1:**
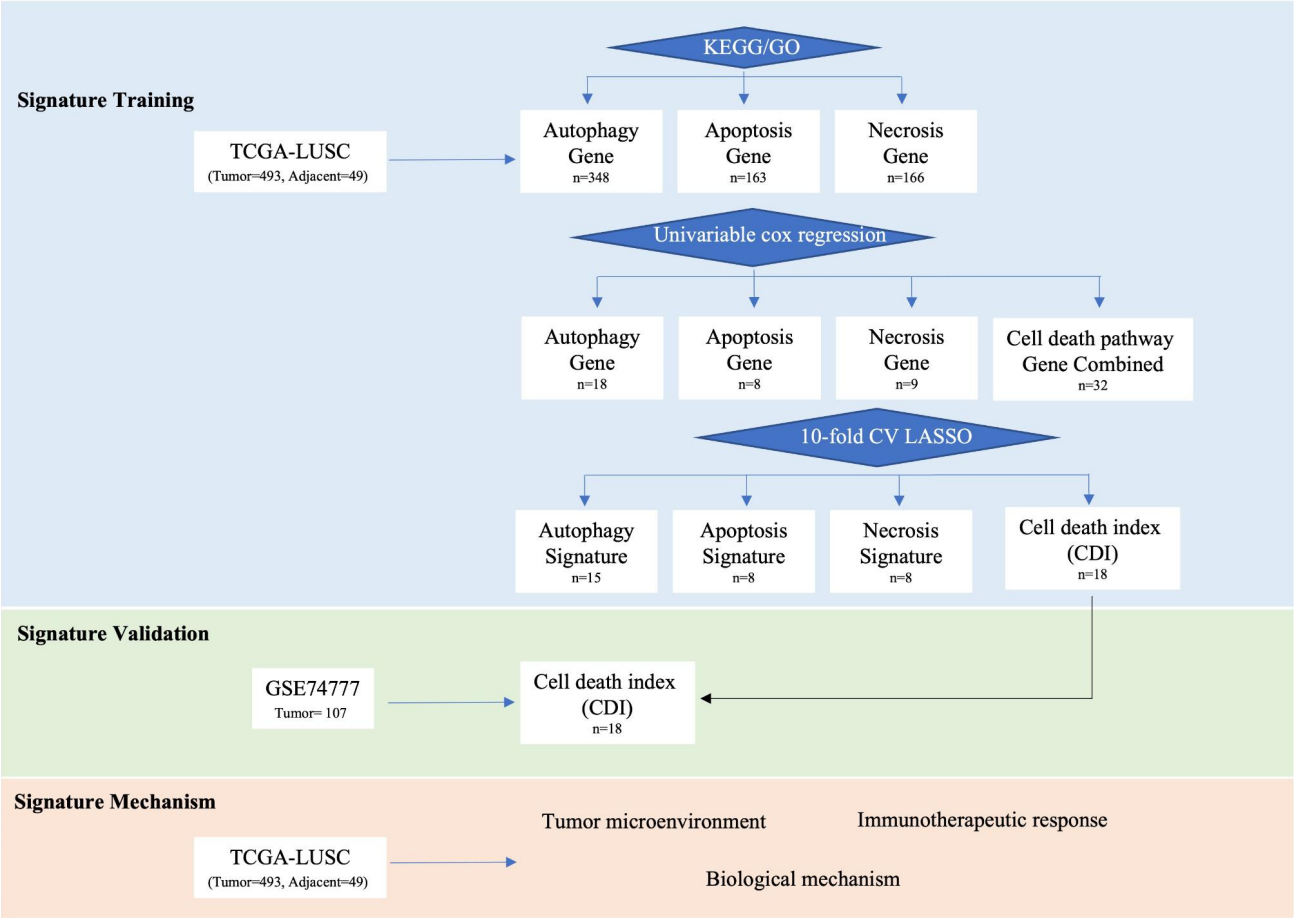
Flow chart of study design

**Fig. 2:**
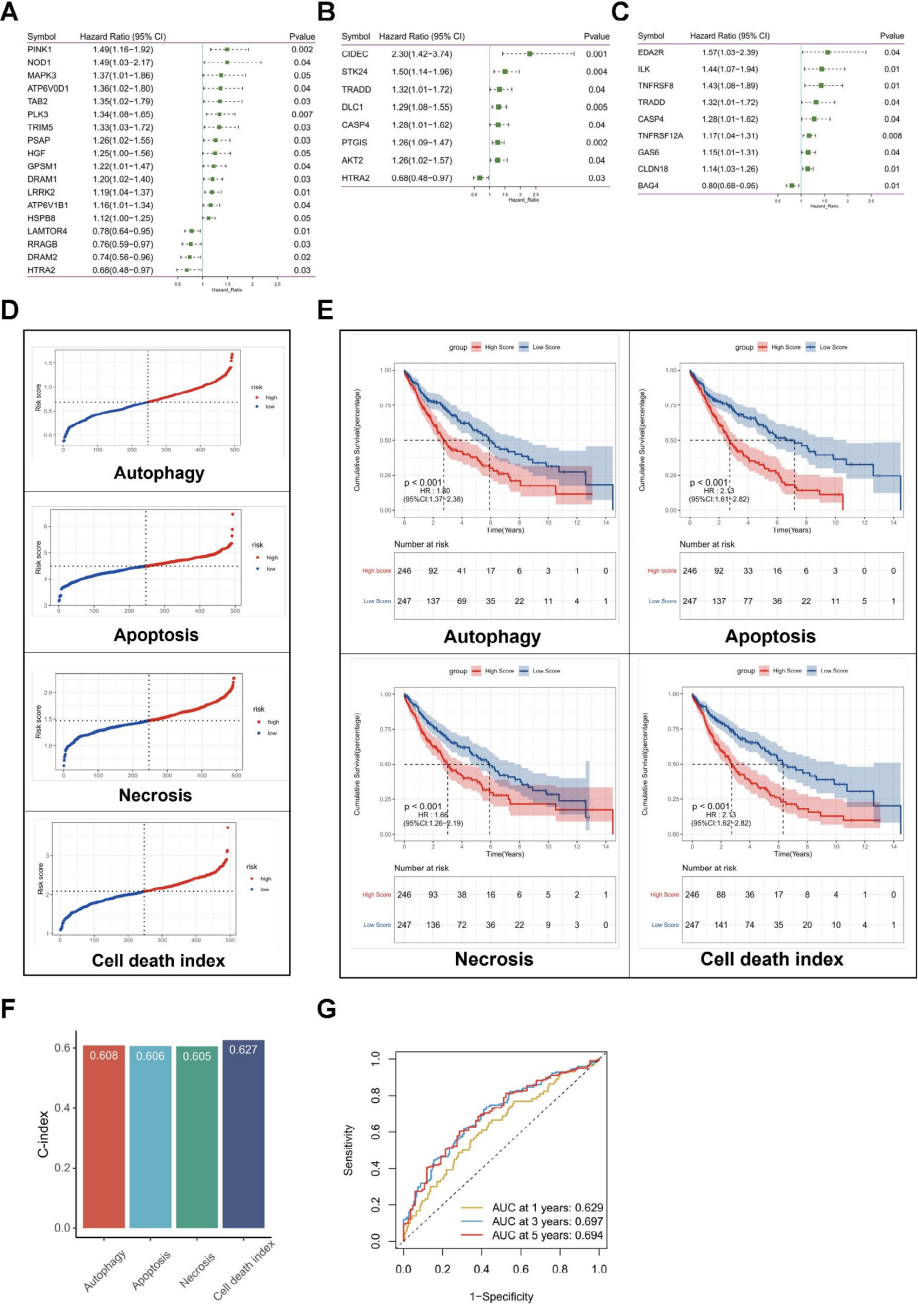
Identification of cell death-associated prognostic signatures for LUSC patients. **(A)** Univariable cox regression analysis between autophagy-related genes and OS in LUSC patients. **(B)** Univariable Cox regression analysis between apoptosis-related genes and OS in LUSC patients. **(C)** Univariable Cox regression analysis between necrosis-related genes and OS in LUSC patients. **(D)** The median value and distribution of the risk scores in the four signatures of autophagy, apoptosis, necrosis pathways, and CDI. **(E)** Kaplan-Meier survival analysis of the four cell death-associated prognostic signatures in the TCGA-LUSC cohort. **(F)** Comparison of the predictive accuracy among the four signatures using the C-index. **(G)** Time-dependent ROC curves analysis of the CDI signature at 1, 3, and 5 years in the TCGA-LUSC cohort

The overall design of this study is shown in the flow diagram (Fig. 1). This study consisted of 493 LUSC samples from the TCGA-LUSC cohort and 107 LUSC samples from the GSE74777 cohort. To generate a prognostic model for LUSC, the univariable Cox analysis was first performed in the TCGA-LUSC cohort to screen the prognostic genes related to cell death (Table S3‒5). There are 18 genes in the autophagy pathway, 8 genes in the apoptosis pathway, and 9 genes in the necrosis pathway with prognostic values (*P* < 0.05, Fig. 2A‒C; Figure S1). Four prognostic signatures for autophagy, apoptosis, and necrosis pathways, and the combination of cell death genes selected in the three pathways were constructed by LASSO cox regression. The optimal value of λ and lists of coefficients of every signature were summarized in Table 1 (Figure S2). The signature of the combined genes was defined as cell death index (CDI). The risk score of the CDI signature was calculated using the following formula: Risk Score = 0.003 × ATP6V0D1 + 0.101 × ATP6V1B1 − 0.052 × DRAM2 + 0.003 × GPSM1 + 0.059 × LRRK2 + 0.052 × MAPK3 + 0.053 × PINK1 − 0.157 × RRAGB + 0.046 × AKT2 + 0.433 × CIDEC − 0.221 × HTRA2 + 0.097 × PTGIS + 0.275 × STK24 − 0.110 × BAG4 + 0.023 × CASP4 + 0.041 × TNFRSF12A + 0.151 × TNFRSF8 + 0.040 × TRADD. The risk scores of other signatures were calculated similarly with their own genes and coefficients. Patients with LUSC were divided into high- and low-risk groups according to the median cutoff value of the risk score (Fig. 2D; Table S6). All four signatures could differentiate patients with different survival (*P* < 0.05, Fig. 2E).

**Table 1:** The prognostic genes and their corresponding coefficients of four cell death signatures

Autophagy		Apoptosis		Necrosis		Cell death index*	
Gene	Coefficient	Gene	Coefficient	Gene	Coefficient	Gene	Coefficient
***ATP6V0D1***	0.061855	***AKT2***	0.158365	***BAG4***	−0.13201	*ATP6V0D1*	0.002993
***ATP6V1B1***	0.122063	*CASP4*	0.124322	***CASP4***	0.056541	*ATP6V1B1*	0.101445
***DRAM2***	−0.07345	***CIDEC***	0.577543	*CLDN18*	0.069005	*DRAM2*	−0.05243
***GPSM1***	0.123344	*DLC1*	0.106105	*EDA2R*	0.002689	*GPSM1*	0.002896
*HGF*	0.031335	***HTRA2***	−0.34211	*ILK*	0.057365	*LRRK2*	0.059429
*HTRA2*	−0.33536	***PTGIS***	0.144802	***TNFRSF12A***	0.081038	*MAPK3*	0.052099
*LAMTOR4*	−0.0163	***STK24***	0.34035	***TNFRSF8***	0.246743	*PINK1*	0.052535
***LRRK2***	0.063269	*TRADD*	0.249061	***TRADD***	0.076235	*RRAGB*	−0.1565
***MAPK3***	0.153165					*AKT2*	0.045682
***PINK1***	0.094258					*CIDEC*	0.432594
*PLK3*	0.037796					*HTRA2*	−0.22073
*PSAP*	0.051802					*PTGIS*	0.096722
***RRAGB***	−0.24185					*STK24*	0.27515
*TAB2*	0.078248					*BAG4*	−0.10963
*TRIM5*	0.022462					*CASP4*	0.023206
						*TNFRSF12A*	0.041075
						*TNFRSF8*	0.150757
						*TRADD*	0.040029

*Cell death index consists of 8 autophagy-related genes (*ATP6V0D1*, *ATP6V1B1*, *DRAM2*, *GPSM1*, *LRRK2*, *MAPK3*, *PINK1*, and *RRAGB*), 5 apoptosis-related genes (*AKT2*, *CIDEC*, *HTRA2*, *PTGIS*, and *STK24*), and 5 necrosis-related genes (*BAG4*, *CASP4*, *TNFRSF12A*, *TNFRSF8*, and *TRADD*), which are highlighted in bold in the three pathways

**Fig. 3:**
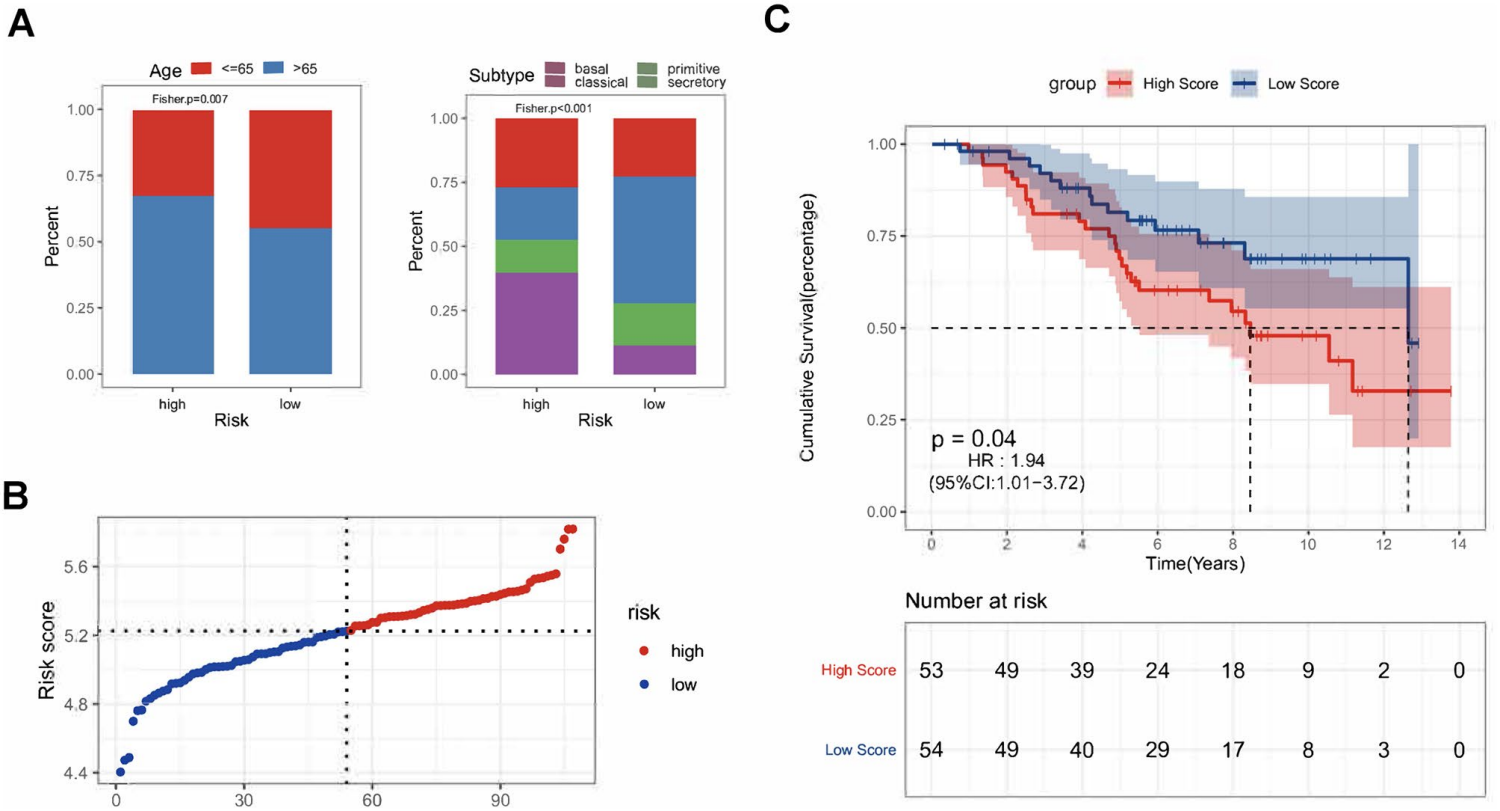
Clinical variable comparisons and validation of the predictive performance of the CDI signature. **(A)** Comparison of the percentage of different age and subtypes of LUSC between high- and low-risk groups by Fisher’s exact test in the TCGA-LUSC cohort. **(B)** The median value and distribution of the risk scores of CDI signature in the GSE74777 cohort. **(C)** Kaplan-Meier survival analysis of the CDI signature of LUSC patients in the GSE74777 cohort

To assess the predictive performance among the four prognostic signatures, the C-index of each signature was calculated. The CDI had the highest C-index (*P* = 0.92, Fig. 2F). Although no significant difference was observed among the four signatures in the C-index analysis, given that the CDI had the highest hazard ratio (same as apoptosis signature, HR, 2.13; 95% CI, 1.61‒2.82; *P* < 0.001, Fig. 2E) and was constructed with genes combining all three pathways, which reflected cell death process more comprehensively, the CDI signature will be further investigated. ROC analysis of the CDI showed that the AUC values of 1-, 3-, and 5-year OS were 0.629, 0.697, and 0.694, respectively, in the TCGA-LUSC cohorts (Fig. 2G). In addition, the age and subtype of LUSC patients were significantly different between the high- and low-risk groups of the CDI (Fig. 3A). There was a higher percentage of LUSC patients with older age in the high-risk group than in the low-risk group. LUSC patients in the high-risk group also presented more basal and secretory subtypes, while those in the low-risk group showed more primitive and classical subtypes.

Moreover, univariable and multivariable cox regression analyses were conducted in the training cohort to explore whether the CDI could be an independent prognostic predictor for OS for LUSC patients. Univariable cox regression analysis showed that both the stage of cancer and the CDI were associated with OS. (stage HR, 1.57; 95% CI, 1.14‒2.16; *P* = 0.006; CDI HR, 2.13; 95% CI, 1.62‒2.82; *P* < 0.001; Table 2). The association between the CDI signature and OS remained significant (HR, 2.10; 95% CI, 1.59‒2.78; *P* < 0.001; Table 2) in the multivariable cox regression after adjusting tumor stage, suggesting that the CDI is an independent prognostic factor for LUSC.

**Table 2:** Univariable and multivariable Cox regression analyses of the CDI in the TCGA-LUSC cohort

	Univariable analysis				Multivariable analysis			
	TCGA-LUSC cohort		GSE74777 cohort		TCGA-LUSC cohort		GSE74777 cohort	
Parameter	**HR** **(95% CI)**	***P *** **value**	**HR** **(95% CI)**	***P *** **value**	**HR** **(95% CI)**	***P *** **value**	**HR** **(95% CI)**	***P *** **value**
Age (> 65 vs. ≤ 65)	1.26 (0.95‒1.69)	0.11	1.39 (0.74‒2.59)	0.3				
Gender (Male vs. Female)	1.19 (0.87‒1.64)	0.28	3.04 (0.73‒12.62)	0.13				
Stage (III/IV vs. I/II)	1.57 (1.14‒2.16)	0.006	1.28 (0.68‒2.39)	0.44	1.52 (1.10‒2.09)	0.01	1.48 (0.79‒2.77)	0.22
CDI (High vs. Low)	2.13 (1.62‒2.82)	< 0.001	1.94 (1.01‒3.72)	0.05	2.10 (1.59‒2.78)	< 0.001	2.01 (1.05‒3.87)	0.05

### Validation of the cell death index as an independent prognostic factor in the validation cohort

To validate the prognostic value of the CDI, 107 LUSC patients from the GSE74777 cohort were analyzed. The 107 LUSC patients were stratified into the high- and low-risk groups according to the risk scores of the CDI signature (Fig. 3B, Table S7). Similar to the results obtained from the TCGA-LUSC cohort, patients in the high-risk group had worse overall survival than those in the low-risk group (HR, 1.94; 95% CI, 1.01‒3.72; *P* = 0.04, Fig. 3C).

We further conducted univariable and multivariable Cox regression to analyze the association of the clinical variables and CDI with OS in validation cohorts. Similar to the training cohort, the CDI signature was associated with OS in the univariable cox regression (HR, 1.94; 95% CI, 1.01‒3.72; *P* = 0.05; Table 2) and in multivariable cox regression (HR, 2.01; 95% CI, 1.05‒3.87; *P* = 0.05; Table 2), suggesting the robustness of the CDI as a prognostic biomarker for LUSC.

### Identification of the biological mechanism and function related to the cell death index

**Fig. 4:**
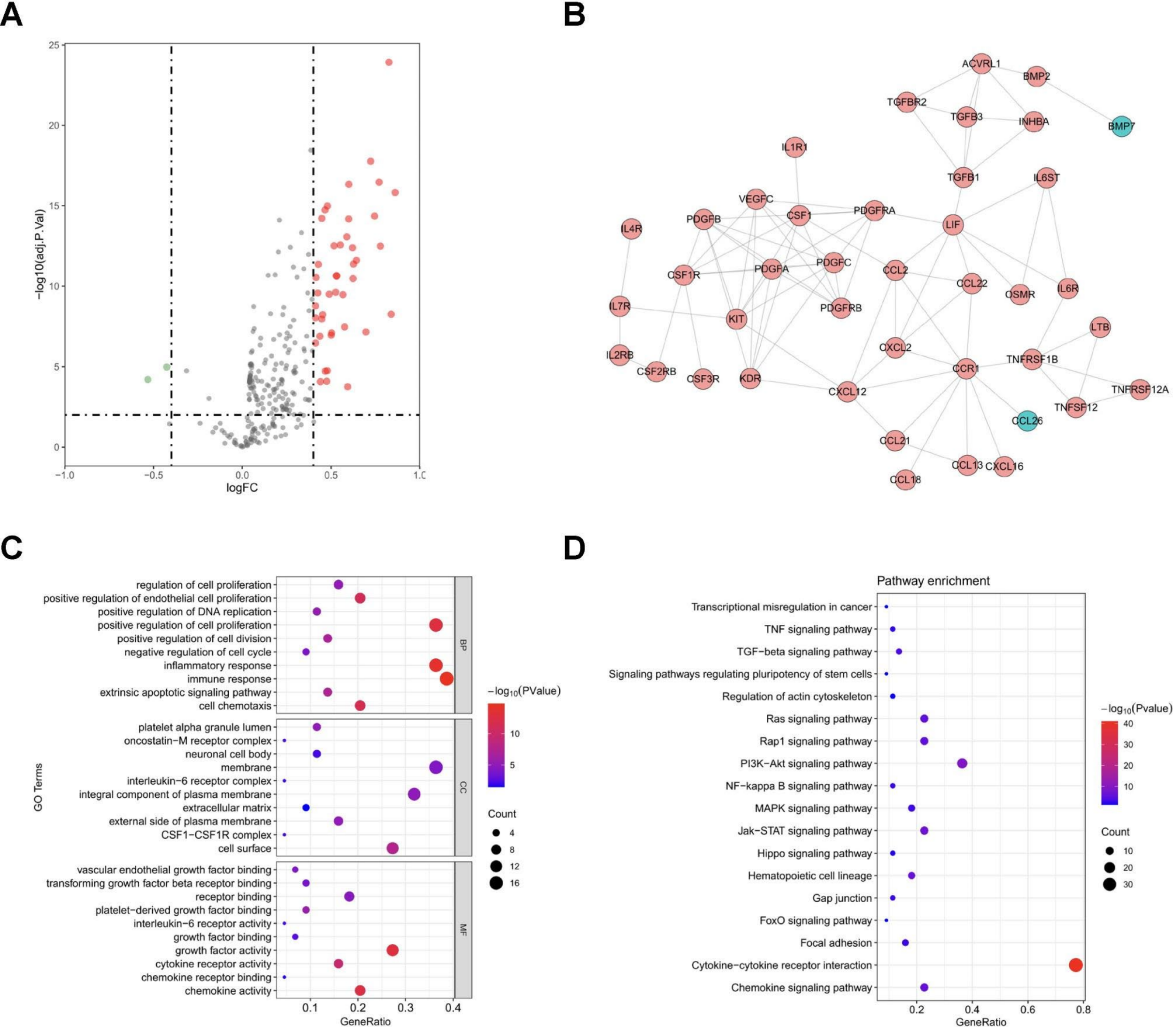
The differentially expressed cytokines and their relevant pathways between high- and low-risk groups of CDI. **(A)** The differentially expressed cytokines between high- and low-risk groups of the CDI. The red dot represents upregulated genes and the green dot for downregulated genes. **(B)** The PPI analysis was performed by all 44 differentially expressed cytokines using online STRING tool. **(C)** GO analyses of the 44 differentially expressed cytokines between high- and low-risk groups. **(D)** KEGG analysis of the 44 differentially expressed cytokines between high- and low-risk groups

To investigate the potential mechanisms involved in the distinct features between high- and low-risk CDI groups, the expressions of 263 cytokines were compared and their biological functions were further analyzed. There were 44 differentially expressed genes (DEGs) of cytokines between the two groups, 2 downregulated and 42 upregulated in the high-risk group (Fig. 4A; Table S8). By using the online STRING tool, we established the PPI network of the DEGs, showing hub genes and their close connection (Fig. 4B). GO analysis revealed that several cell death-associated and immune-related pathways were enriched, such as inflammatory response, immune response, extrinsic apoptotic signaling pathway (Fig. 4C). KEGG terms showed that the DEGs were enriched in biological pathways of the tumor initiation, progression, and stemness, including TNF signaling pathway, TGF-beta signaling pathway, and signaling pathways regulating pluripotency of stem cells and actin cytoskeleton (Fig. 4D).

### Correlation between cell death index and tumor microenvironment and stemness

**Fig. 5:**
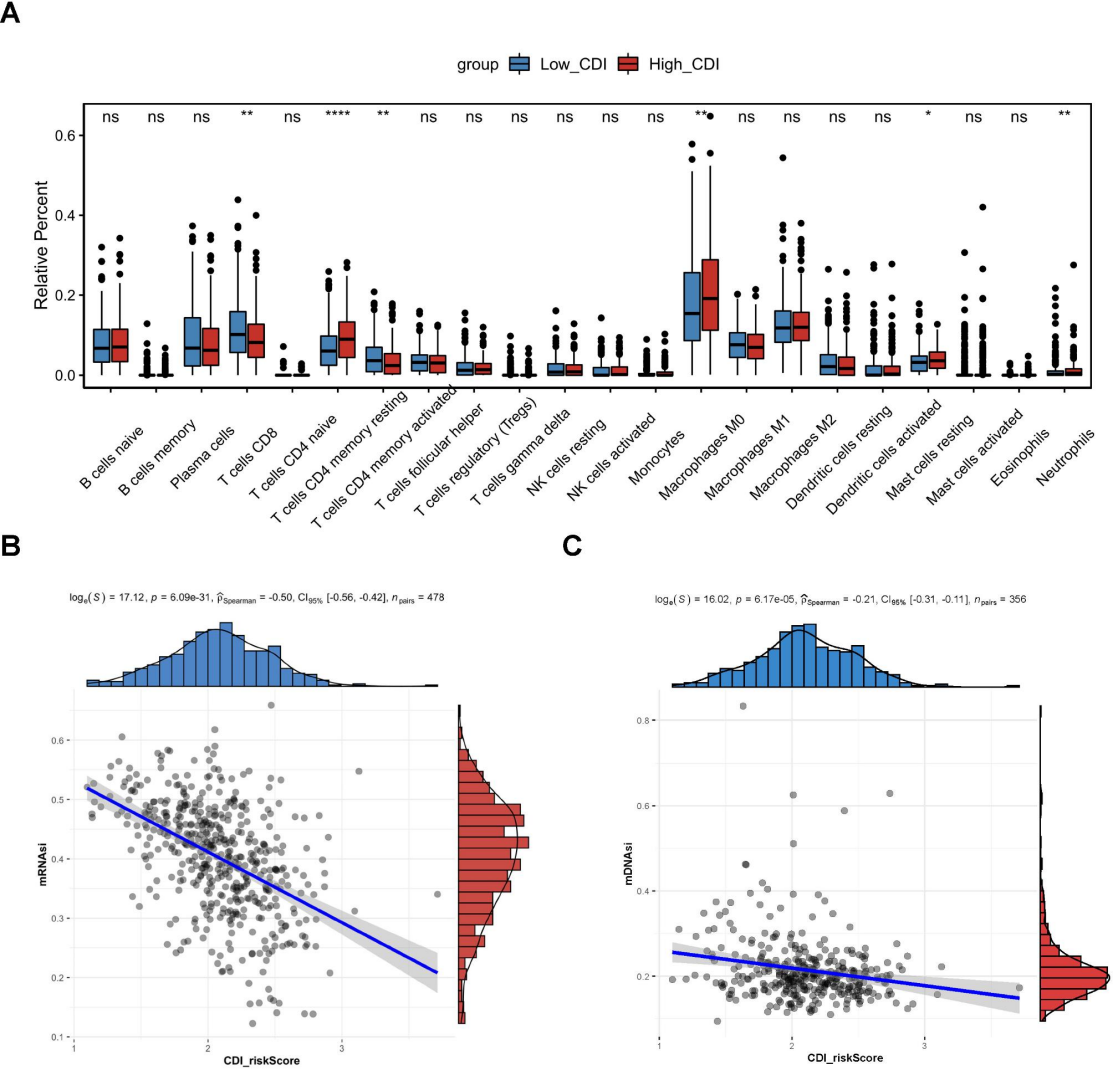
Association between the risk score with immune cell infiltration and tumor stemness. **(A)** Comparison of the infiltration fraction of 22 types of immune cells between the high- and low-risk groups of CDI signature. **(B)** Spearman correlation analysis between the risk score of the CDI and mRNAsi. **(C)** Spearman correlation analysis between the risk score of the CDI and mDNAsi

As one of the important components of TME, the distribution of immune cells closely correlates with immunotherapy strategies for patients with lung cancers. The contents of immune cells were significantly different between high- and low-risk scores of CDI in LUSC, suggesting different immune statuses in the two groups (Fig. 5A). Naive CD4^+^ T cells, monocytes, activated dendritic cells (DCs), and neutrophils were higher in the high-risk group compared with the low-risk group (*P* < 0.05, Fig. 5A). Meanwhile, the fractions of plasma cells and resting memory CD4^+^ T cells were significantly higher in the low-risk group than those in the high-risk group (*P* < 0.05, Fig. 5A).

We then analyzed the relationship between the risk score of CDI and cancer stem cells, which can influence tumor immunogenicity and susceptibility to immunotherapy. mRNAsi and mDNAsi are two independent indices to quantify the degree of differentiation and stemness of LUSC. The mRNAsi and mDNAsi of 33 cancer types in the TCGA have been previously studied and reported [29], where we retrieved mRNAsi and mDNAsi data of the TCGA-LUSC cohort. Then, we analyzed the correlation between CDI and two indices. In the training cohort, both mRNAsi (Fig. 5B) and mDNAsi (Fig. 5C) were significantly negatively correlated with the risk score of CDI signature. Given the significant correlation between CDI and tumor microenvironment, this signature might be promising and reliable for predicting immunotherapeutic response.

### Cell death index might be an indicator to predict immunotherapeutic outcomes in LUSC

**Fig. 6:**
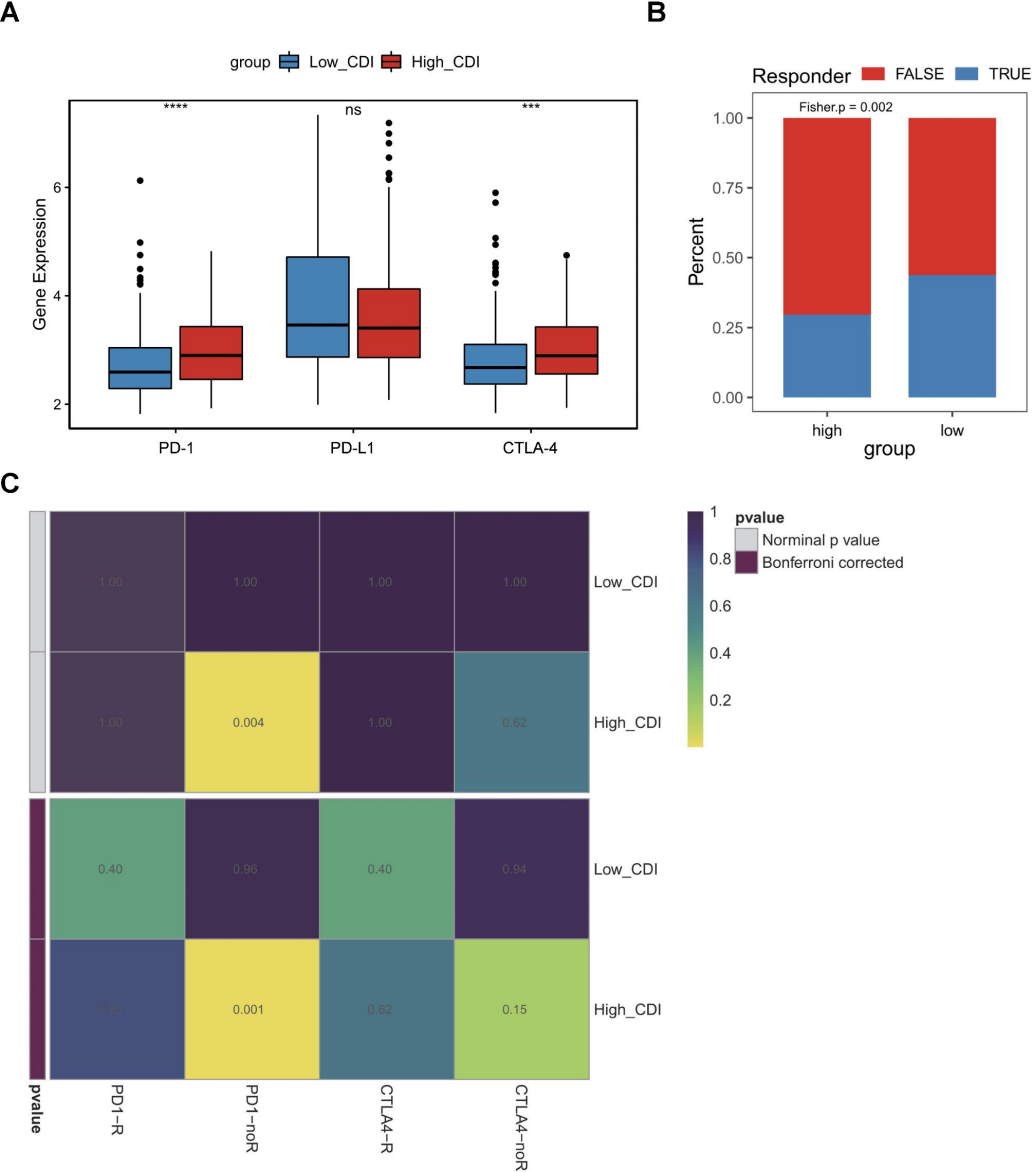
Association between the CDI signature and different likelihoods of immunotherapeutic response. **(A)** Comparison of the expression of immune checkpoint molecules, namely PD-1, PD-L1, and CTLA4 between high- and low-risk groups of CDI signature. **(B)** Comparison of the percentage of the responders to immunotherapy between high- and low-risk groups of CDI. TIDE algorithm was used to predict the likelihood of immunotherapeutic response. **(C)** Comparison of the likelihood of the response to immunotherapy between high- and low-risk groups of CDI. Submap analysis was used to predict the likelihood of immunotherapeutic response. R, response.

As key regulators in immune escape, the expression levels of immune checkpoint molecules such as PD-1, PD-L1, and cytotoxic T-lymphocyte associated protein 4 (CTLA-4) are important indicators for individualized immunotherapy. We found that patients of LUSC in the high-risk group of the CDI signature had a higher gene expression of PD-1 and CTLA-4 than patients in the low-risk group. Yet, no significant difference in PD-L1 was detected between the two groups (Fig. 6A). We further predicted the likelihood of immunotherapeutic response in LUSC patients with TIDE analysis. The response rate to immunotherapy in LUSC was significantly different between the two groups. According to the TIDE results, more LUSC patients would likely to respond to ICI treatments in the low-risk group of the CDI signature than in the high-risk group (*P* = 0.002, Fig. 6B). The SubMap analysis also demonstrated that patients in the high-risk group exhibited a similar gene expression profile to LUSC patients who did not respond to ICIs (*P* = 0.001, Fig. 6C). Thus, patients in the low-risk group may be potential candidates for receiving immunotherapy.

## Discussion

Most patients with LUSC are diagnosed at an advanced stage and consequently, present unfavorable therapeutic effects and poor prognoses [30]. Even with surgery and other treatments, the 5-year survival rate of patients is only about 15%. The emergence of ICIs, such as PD-1/PD-L1- and CTLA4-targeted therapy, has revolutionized the standard of care for LUSC patients. Although immunotherapy is a promising cancer treatment approach, only about 20% of NSCLC patients respond to anti-PD-1/PD-L1 therapy [31]. Due to the lack of effective biomarkers, prognostic stratification and predictive identification of LUSC patients who are sensitive to immunotherapy is challenging [32]. Here, we construct a cell death-associated signature, CDI, consisting of 18 genes for LUSC patients and determined the prognostic value and reliable ability for predicting immunotherapeutic response. Patients in the high-risk group of the CDI signature had significantly worse survival outcome than those in the low-risk group. In addition, we revealed a significant relationship between CDI signature and infiltrated immune cells as well as tumor stemness indices. TIDE prediction analysis also showed that more patients in the low-risk group of the CDI signature would be more likely to respond to the immunotherapy, suggesting that if patients in the low-risk group of the CDI signature of a cohort, immunotherapy might be a potential treatment.

Autophagy, apoptosis, and necrosis are three classical cell death pathways, which assist in tumor growth, metastasis, and drug resistance [6] and can serve as novel biomarkers and potential therapeutic alternatives for LUSC. A previous study indicated that an autophagy-related signature had good performance for predicting the survival of LUAD and LUSC patients with a better AUC than other clinical parameters [33]. However, cell death-related signatures for LUSC are still lacking, and the existing prognostic models either lack the association with the immune microenvironment or the prediction of immunotherapeutic response. Compared with these studies, we combined genes associated with autophagy, apoptosis, and necrosis to establish a prognostic signature. This CDI signature predicted the prognosis of patients with LUSC better than other signatures of cell death pathways. We further analyzed the relationship of CDI signature with clinical characteristics and tumor microenvironment. We found that the CDI signature is an independent predictive prognostic factor and distinguishes the distinct immune status of LUSC, indicating its potential as a useful tool to select immunotherapy-sensitive patients.

The majority of the cell death-related genes in the CDI signature are known to be involved in LUSC progression as potential prospective biomarkers and therapeutic targets. For instance, ALK rearrangement involving ATPase H + transporting V1 subunit B1 (ATP6V1B1) [34] and Bcl-2-associated athanogene 4 (BAG4)-fibroblast growth factor receptor 1 (FGFR1) fusion [35] were detected in NSCLC. ATP6V1B1 encodes a component of vacuolar ATPase (V-ATPase). Decreased V-ATPase activity changes the cytoplasmic pH of cancer cells to repress granzyme bioactivity, which adversely influences the apoptosis of cancer cells induced by NK cells [36]. As a member of the Roco protein family, leucine-rich repeat kinase 2 (LRRK2) is frequently mutated in LUSC patients and involved in the metastasis and prognosis of LUSC [37, 38]. Damage-regulated autophagy modulator 2 (DRAM2) [39], caspase-4, which is encoded by CASP4 [40], and fibroblast growth factor-inducible 14 (Fn14; TNFRSF12A) [41] were frequently overexpressed in NSCLC tissues. A higher expression of DRAM2 may promote cell metastasis and proliferation by reducing the expression of p53 [39]. Caspase-4 positive LUAD (79.3%) and LUSC (88.2%) patients have poorer survival compared with patients with lower levels of caspase-4 [40]. Prostaglandin I2 synthase (PTGIS) is a key regulator in the synthesis of prostaglandin I2, which plays multiple roles in inflammation and immune modulation. The level of PTGIS was lower in lung cancer compared with the normal tissues and is associated with a variety of immune markers and the survival of patients with lung cancer [42]. Downregulation of miR-497-targeted AKT2 might affect tumor growth and chemoresistance to cisplatin in NSCLC [43]. In addition, the role of several genes that have been reported in other types of cancers remains to be explored in LUSC prognosis, such as mitogen-activated protein kinase 2 (MAPK2) [44], G protein signaling modulator 1 (GPSM1) [45, 46], tumor necrosis factor receptor superfamily member 8 (TNFRSF8; CD30) [47] and TNF receptor type 1 associated death domain protein (TRADD) [48].

TME is a complex and dynamic ecosystem composed of diverse cell types including cancer stem cells and infiltrating immune cells [49]. This ecosystem regulates the production and bioactivity of growth factors, inflammatory factors, cytokines, and immune components to create a fertile soil for tumor initiation and progression, and influences the response of therapy [50, 51]. In this study, we observed significant differences in immune response and stemness-associated signaling pathways between the two risk groups, through the enrichment analysis of DEGs. Those pathways (e.g. vascular endothelial growth factor, transforming growth factor β, Ras, PI3K-AKT, NF-κB, JAK-STAT, and Hippo) play critical roles in the dedifferentiation, proliferation, and metastasis of LUSC, as well as the development of many other human diseases [51]. While our study focused on the LUSC, the importance of cell death might be worth investigating in other types of cancer and autoimmune diseases.

The innate and adaptive immune cells in the lung TME are double-edged for patients and may help predict prognosis and therapy outcome [52]. Importantly, tumor cells can accumulate mutations, acquire properties, and remodel the surrounding microenvironment, which allows them to evade the surveillance of the host immune system, such as the increase of immunosuppressive cells, the decrease of immunoreactive cells and elevated expression of immunosuppressive molecules [53]. Therefore, we speculated that patients in different risk groups might present different immune cell infiltration and immunotherapeutic responses. As expected, the higher levels of activated DCs and neutrophils in the high-risk group than in the low-risk group. DCs can upregulate the co-inhibitory molecule B7-H3 (also known as CD276) and thus reduce the stimulation of T cells in patients with NSCLC [54]. In NSCLC, neutrophils dominate the immune landscape, and their depletion is associated with a marked reduction in lung carcinogenesis [51]. In addition, a subset of tumor-associated neutrophils that exhibit characteristics of both neutrophils and antigen-presenting cells in early-stage human lung cancer has been proven to augment antitumor T cell response [55]. Consistently, TIDE prediction and Submap analysis demonstrated that LUSC patients in the low-risk group were more likely to respond to immunotherapy. These findings indicate that CDI signature may help tailor personalized immunotherapy for LUSC patients in the future.

However, certain limitations in our study should be considered. Firstly, it was a retrospective study based on data from public datasets, some information may not be available. Secondly, in addition to transcriptome data used in this study, other omics data such as proteome and metabolome can be used to create a more precise model for tumor diagnosis and prognosis. Thirdly, the in-depth molecular mechanisms by which cell death-related genes affect LUSC progression require verification by in vitro and in vivo experiments.

## Conclusions

In summary, our study constructed a cell death-associated signature, CDI, which is an independent prognostic indicator able to predict the prognosis for LUSC patients. Furthermore, patients at lower risk might benefit from the PD-1 or CTLA-4 blockade. With further validation, this signature could be developed as a prognostic and predictive biomarker for precise and personalized therapies for LUSC patients.

## Electronic supplementary material

Below is the link to the electronic supplementary material.

Supplementary Material 1

Supplementary Material 2

## Data Availability

The datasets generated and/or analyzed during the current study are available in the TCGA database (TCGA-LUSC) and the GEO database (GSE74777).

## Abbreviations

LUSC: Lung squamous cell carcinoma
TCGA: The Cancer Genome Atlas
GEO: Gene Expression Omnibus
KEGG: Kyoto Encyclopedia of Genes and Genomes
GO: Gene Ontology
CDI: Cell death index
NSCLC: Non-small cell lung cancer
AJCC: American Joint Committee on Cancer
Caspases: Cysteinyl aspartate proteases
TME: Tumor microenvironment
ICIs: Immune checkpoint inhibitors
PD-1: Programmed cell death 1
PD-L1: Programmed cell death-ligand 1
LUAD: Lung adenocarcinoma
TPM: Transcript per million
LASSO: Least absolute shrinkage and selection operator
C-index: Concordance index
ROC: Receiver-operating characteristic
DEGs: Differentially expressed genes
FDR: False discovery rate
DAVID: Database for Annotation, Visualization and Integrated Discovery
PPI: Protein-protein interaction
STRING: Search Tool for the Retrieval of Interacting Genes
CIBERSORT: Cell Type Identification by Estimating Relative Subsets of RNA Transcripts
TIDE: Tumor Immune Dysfunction and Exclusion
Submap: Subclass mapping
AUC: Area under the curve
OS: Overall survival
DCs: Dendritic cells
CTLA-4: Cytotoxic T-lymphocyte-associated protein 4
ATP6V1B1: ATPase H + transporting V1 subunit B1
BAG4: Bcl-2-associated athanogene 4
FGFR1: Fibroblast growth factor receptor 1
V-ATPase: Vacuolar ATPase
LRRK2: Leucine-rich repeat kinase 2
DRAM2: Damage-regulated autophagy modulator 2
Fn14: Fibroblast growth factor-inducible 14
PTGIS: Prostaglandin I2 synthase
MAPK2: Mitogen-activated protein kinase 2
GPSM1: G protein signaling modulator 1
TNFRSF8: Tumor necrosis factor receptor superfamily member 8
TRADD: TNF receptor type 1 associated death domain protein

## References

[1] Sung H, Ferlay J, Siegel RL, Laversanne M, Soerjomataram I, Jemal A, Bray F (2021). Global Cancer Statistics 2020: GLOBOCAN estimates of incidence and mortality worldwide for 36 cancers in 185 countries. Cancer J Clin.

[2] Siegel RL, Miller KD, Fuchs HE, Jemal A (2022). Cancer statistics, 2022. Cancer J Clin.

[3] Youlden DR, Cramb SM, Baade PD (2008). The international epidemiology of lung cancer: geographical distribution and secular trends. J Thorac oncology: official publication Int Association Study Lung Cancer.

[4] Lancia A, Merizzoli E, Filippi AR. The 8(th) UICC/AJCC TNM edition for non-small cell lung cancer staging: getting off to a flying start? Annals of translational medicine 2019, 7(Suppl 6):S205. 10.21037/atm.2019.07.02 PMC6789332 31656784

[5] Shi S, Xie H, Yin W, Zhang Y, Peng X, Yu F, Shemanski KA, Kim AW, Wang X (2020). The prognostic significance of the 8th edition AJCC TNM staging system for non-small-cell lung cancer is not applicable to lung cancer as a second primary malignancy. J Surg Oncol.

[6] Hotchkiss RS, Strasser A, McDunn JE, Swanson PE (2009). Cell death. N Engl J Med.

[7] Shi Y (2002). Mechanisms of caspase activation and inhibition during apoptosis. Mol Cell.

[8] Mizushima N (2007). Autophagy: process and function. Genes Dev.

[9] Qu X, Zou Z, Sun Q, Luby-Phelps K, Cheng P, Hogan RN, Gilpin C, Levine B (2007). Autophagy gene-dependent clearance of apoptotic cells during embryonic development. Cell.

[10] Colombo MI (2007). Autophagy: a pathogen driven process. IUBMB Life.

[11] Mathew R, Karantza-Wadsworth V, White E (2007). Role of autophagy in cancer. Nat Rev Cancer.

[12] Galluzzi L, Kroemer G (2008). Necroptosis: a specialized pathway of programmed necrosis. Cell.

[13] Nagata S, Tanaka M (2017). Programmed cell death and the immune system. Nat Rev Immunol.

[14] Lei X, Lei Y, Li JK, Du WX, Li RG, Yang J, Li J, Li F, Tan HB (2020). Immune cells within the tumor microenvironment: Biological functions and roles in cancer immunotherapy. Cancer Lett.

[15] Paz-Ares L, Luft A, Vicente D, Tafreshi A, G¨¹m¨¹ M, Mazi¨¨res J, Hermes B, ay ?enler F, Cs?szi T, F¨¹l?p A, et al. Pembrolizumab plus chemotherapy for squamous non-small-cell lung cancer. N Engl J Med. 2018;379(21):2040–51. 10.1056/NEJMoa1810865 30280635

[16] Brahmer J, Reckamp KL, Baas P, Crin¨° L, Eberhardt WE, Poddubskaya E, Antonia S, Pluzanski A, Vokes EE, Holgado E (2015). Nivolumab versus docetaxel in advanced squamous-cell non-small-cell lung cancer. N Engl J Med.

[17] Rittmeyer A, Barlesi F, Waterkamp D, Park K, Ciardiello F, von Pawel J, Gadgeel SM, Hida T, Kowalski DM, Dols MC (2017). Atezolizumab versus docetaxel in patients with previously treated non-small-cell lung cancer (OAK): a phase 3, open-label, multicentre randomised controlled trial. Lancet (London England).

[18] Carneiro BA, El-Deiry WS (2020). Targeting apoptosis in cancer therapy. Nat reviews Clin Oncol.

[19] Asano K, Nabeyama A, Miyake Y, Qiu CH, Kurita A, Tomura M, Kanagawa O, Fujii S, Tanaka M (2011). CD169-positive macrophages dominate antitumor immunity by crosspresenting dead cell-associated antigens. Immunity.

[20] Creagh EM, Conroy H, Martin SJ (2003). Caspase-activation pathways in apoptosis and immunity. Immunol Rev.

[21] Zhu CQ, Strumpf D, Li CY, Li Q, Liu N, Der S, Shepherd FA, Tsao MS, Jurisica I (2010). Prognostic gene expression signature for squamous cell carcinoma of lung. Clin cancer research: official J Am Association Cancer Res.

[22] Hu J, Xu L, Shou T, Chen Q (2019). Systematic analysis identifies three-lncRNA signature as a potentially prognostic biomarker for lung squamous cell carcinoma using bioinformatics strategy. Translational lung cancer research.

[23] Li J, Wang J, Chen Y, Yang L, Chen S (2017). A prognostic 4-gene expression signature for squamous cell lung carcinoma. J Cell Physiol.

[24] Martínez-Terroba E, Behrens C, Agorreta J, Mons¨® E, Millares L, Felip E, Rosell R, Ramirez JL, Remirez A, Torre W (2019). 5 protein-based signature for resectable lung squamous cell carcinoma improves the prognostic performance of the TNM staging. Thorax.

[25] Sun S, Yang Y, Yang Z, Wang J, Li R, Tian H, Tan F, Xue Q, Gao Y, He J (2021). Ferroptosis characterization in lung adenocarcinomas reveals prognostic signature with immunotherapeutic implication. Front cell Dev biology.

[26] Ahluwalia P, Ahluwalia M, Mondal AK, Sahajpal N, Kota V, Rojiani MV, Rojiani AM, Kolhe R. Immunogenomic gene signature of cell-death associated genes with prognostic implications in lung cancer.Cancers2021, 13(1). 10.3390/cancers13010155 PMC7795632 33466402

[27] Liu H, Gao L, Xie T, Li J, Zhai TS, Xu Y (2021). Identification and validation of a prognostic signature for prostate cancer based on ferroptosis-related genes. Front Oncol.

[28] Ju A, Tang J, Chen S, Fu Y, Luo Y (2021). Pyroptosis-related gene signatures can robustly diagnose skin cutaneous melanoma and predict the prognosis. Front Oncol.

[29] Van Allen EM, Miao D, Schilling B, Shukla SA, Blank C, Zimmer L, Sucker A, Hillen U, Foppen MHG, Goldinger SM (2015). Genomic correlates of response to CTLA-4 blockade in metastatic melanoma. Science.

[30] Miller KD, Nogueira L, Mariotto AB, Rowland JH, Yabroff KR, Alfano CM, Jemal A, Kramer JL, Siegel RL. Cancer treatment and survivorship statistics, 2019. CA: a cancer journal for clinicians 2019, 69(5):363–385. 10.3322/caac.21565 31184787

[31] Carrizosa DR, Gold KA (2015). New strategies in immunotherapy for non-small cell lung cancer. Translational lung cancer research.

[32] Malhotra J, Jabbour SK, Aisner J (2017). Current state of immunotherapy for non-small cell lung cancer. Translational lung cancer research.

[33] Zhu J, Wang M, Hu D (2020). Development of an autophagy-related gene prognostic signature in lung adenocarcinoma and lung squamous cell carcinoma. PeerJ.

[34] Rosenbaum JN, Bloom R, Forys JT, Hiken J, Armstrong JR, Branson J, McNulty S, Velu PD, Pepin K, Abel H (2018). Genomic heterogeneity of ALK fusion breakpoints in non-small-cell lung cancer. Mod pathology: official J United States Can Acad Pathol Inc.

[35] Wang R, Wang L, Li Y, Hu H, Shen L, Shen X, Pan Y, Ye T, Zhang Y, Luo X (2014). FGFR1/3 tyrosine kinase fusions define a unique molecular subtype of non-small cell lung cancer. Clin cancer research: official J Am Association Cancer Res.

[36] Nishie M, Suzuki E, Hattori M, Kawaguch K, Kataoka TR, Hirata M, Pu F, Kotake T, Tsuda M, Yamaguchi A (2021). Downregulated ATP6V1B1 expression acidifies the intracellular environment of cancer cells leading to resistance to antibody-dependent cellular cytotoxicity. Cancer Immunol immunotherapy: CII.

[37] Meng F, Zhang L, Ren Y, Ma Q (2019). The genomic alterations of lung adenocarcinoma and lung squamous cell carcinoma can explain the differences of their overall survival rates. J Cell Physiol.

[38] Qi L, Gao C, Feng F, Zhang T, Yao Y, Wang X, Liu C, Li J, Li J, Sun C (2019). MicroRNAs associated with lung squamous cell carcinoma: New prognostic biomarkers and therapeutic targets. J Cell Biochem.

[39] Wudu M, Ren H, Hui L, Jiang J, Zhang S, Xu Y, Wang Q, Su H, Jiang X, Dao R (2019). DRAM2 acts as an oncogene in non-small cell lung cancer and suppresses the expression of p53. J experimental Clin cancer research: CR.

[40] Terlizzi M, Colarusso C, De Rosa I, Somma P, Curcio C, Aquino RP, Panico L, Salvi R, Zito Marino F, Botti G (2020). Identification of a novel subpopulation of Caspase-4 positive non-small cell lung Cancer patients. J experimental Clin cancer research: CR.

[41] Whitsett TG, Cheng E, Inge L, Asrani K, Jameson NM, Hostetter G, Weiss GJ, Kingsley CB, Loftus JC, Bremner R (2012). Elevated expression of Fn14 in non-small cell lung cancer correlates with activated EGFR and promotes tumor cell migration and invasion. Am J Pathol.

[42] Dai D, Chen B, Feng Y, Wang W, Jiang Y, Huang H, Liu J (2020). Prognostic value of prostaglandin I2 synthase and its correlation with tumor-infiltrating immune cells in lung cancer, ovarian cancer, and gastric cancer. Aging.

[43] Wang L, Ji XB, Wang LH, Qiu JG, Zhou FM, Liu WJ, Wan DD, Lin MC, Liu LZ, Zhang JY (2020). Regulation of microRNA-497-targeting AKT2 influences tumor growth and chemoresistance to cisplatin in lung cancer. Front cell Dev biology.

[44] Deng R, Zhang HL, Huang JH, Cai RZ, Wang Y, Chen YH, Hu BX, Ye ZP, Li ZL, Mai J (2021). MAPK1/3 kinase-dependent ULK1 degradation attenuates mitophagy and promotes breast cancer bone metastasis. Autophagy.

[45] Zhang Y, Zhou B, Sun J, He Q, Zhao Y (2021). Knockdown of GPSM1 inhibits the proliferation and promotes the apoptosis of B-cell acute lymphoblastic leukemia cells by suppressing the ADCY6-RAPGEF3-JNK signaling pathway. Pathol Oncol research: POR.

[46] Adekoya TO, Smith N, Aladeniyi T, Blumer JB, Chen XL, Richardson RM (2019). Activator of G protein signaling 3 modulates prostate tumor development and progression. Carcinogenesis.

[47] Dabir S, Kresak A, Yang M, Fu P, Wildey G, Dowlati A (2015). CD30 is a potential therapeutic target in malignant mesothelioma. Mol Cancer Ther.

[48] Xu D, Zhao H, Jin M, Zhu H, Shan B, Geng J, Dziedzic SA, Amin P, Mifflin L, Naito MG (2020). Modulating TRADD to restore cellular homeostasis and inhibit apoptosis. Nature.

[49] Hinshaw DC, Shevde LA (2019). The Tumor Microenvironment innately modulates Cancer Progression. Cancer Res.

[50] Binnewies M, Roberts EW, Kersten K, Chan V, Fearon DF, Merad M, Coussens LM, Gabrilovich DI, Ostrand-Rosenberg S, Hedrick CC (2018). Understanding the tumor immune microenvironment (TIME) for effective therapy. Nat Med.

[51] Altorki NK, Markowitz GJ, Gao D, Port JL, Saxena A, Stiles B, McGraw T, Mittal V (2019). The lung microenvironment: an important regulator of tumour growth and metastasis. Nat Rev Cancer.

[52] Gajewski TF, Schreiber H, Fu YX (2013). Innate and adaptive immune cells in the tumor microenvironment. Nat Immunol.

[53] Noro R, Ishigame T, Walsh N, Shiraishi K, Robles AI, Ryan BM, Schetter AJ, Bowman ED, Welsh JA, Seike M (2017). A two-gene prognostic classifier for early-stage lung squamous cell carcinoma in multiple large-scale and geographically diverse cohorts. J Thorac oncology: official publication Int Association Study Lung Cancer.

[54] Schneider T, Hoffmann H, Dienemann H, Schnabel PA, Enk AH, Ring S, Mahnke K (2011). Non-small cell lung cancer induces an immunosuppressive phenotype of dendritic cells in tumor microenvironment by upregulating B7-H3. J Thorac oncology: official publication Int Association Study Lung Cancer.

[55] Singhal S, Bhojnagarwala PS, Brien S, Moon EK, Garfall AL, Rao AS, Quatromoni JG, Stephen TL, Litzky L, Deshpande C (2016). Origin and role of a subset of tumor-associated neutrophils with antigen-presenting cell features in early-stage human lung cancer. Cancer Cell.

