# Supplementary material for: Deciphering a cell death-associated signature for predicting prognosis and response to immunotherapy in lung squamous cell carcinoma
Source: Respir Res. 2023 Jul 6;24:176. doi: 10.1186/s12931-023-02402-9 (PMC10324233; doi:10.1186/s12931-023-02402-9)

**Supplementary Materials**

**Figure S1. Survival analysis of cell death-associated prognostic genes according to the median cutoff expression value.**

**
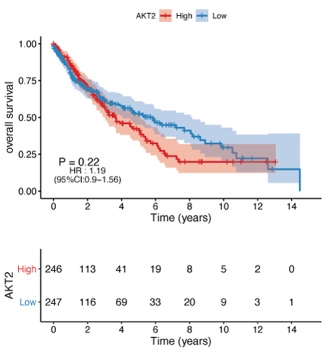

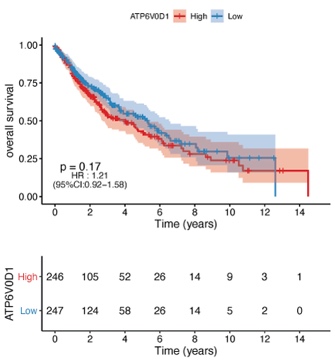

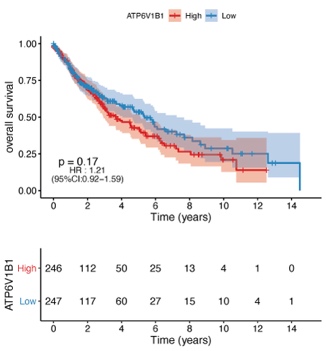

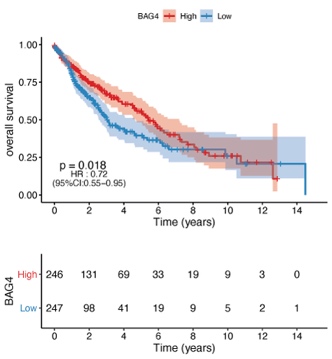
**

**
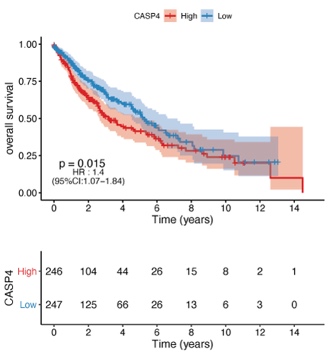

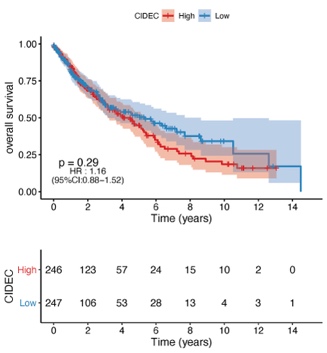

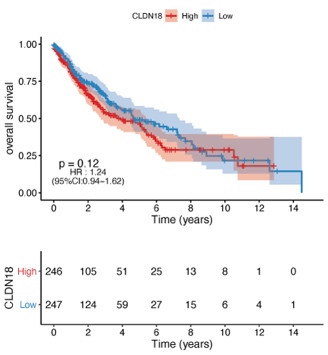

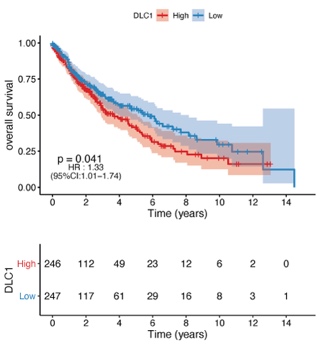
**

**
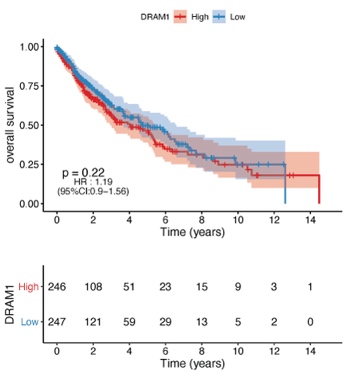

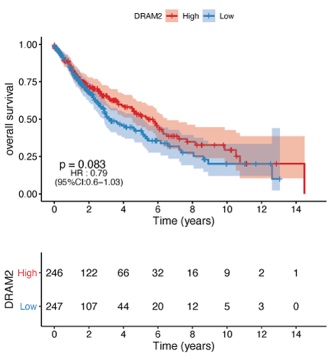

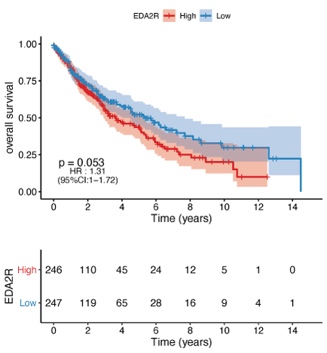

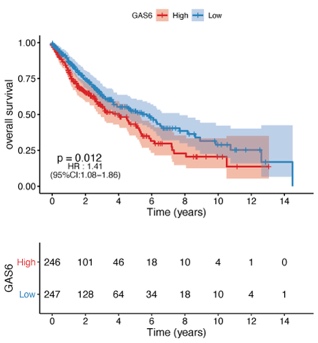
**

**
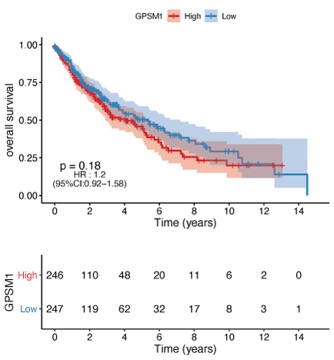

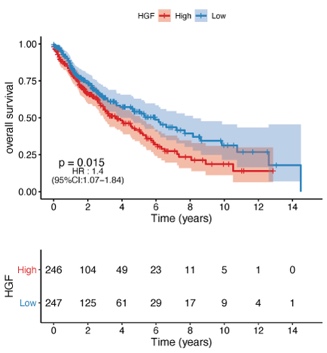

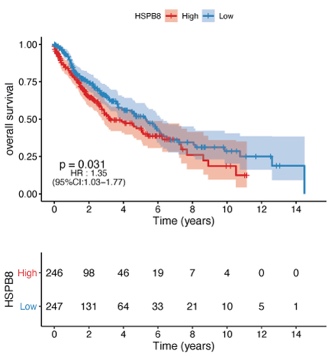

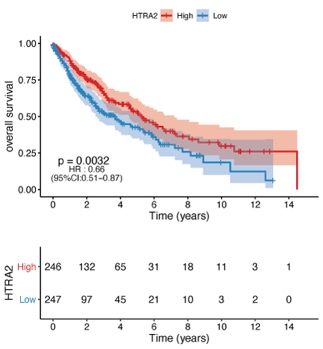
**

**
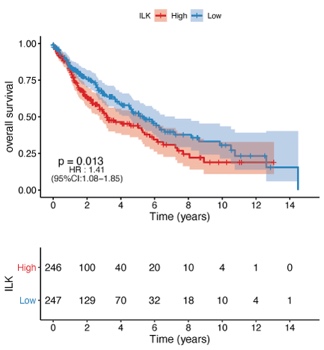

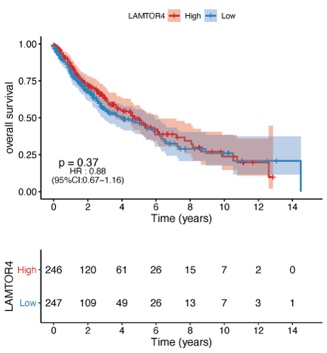

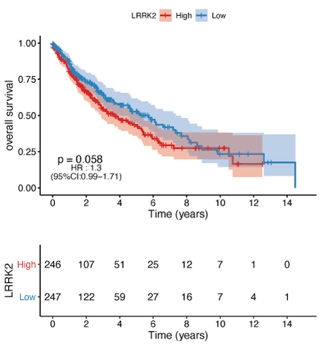

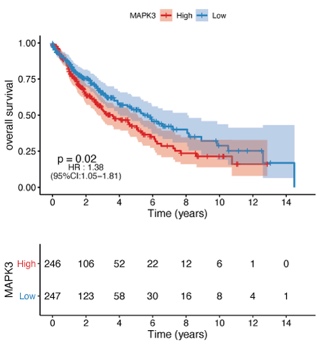
**

**
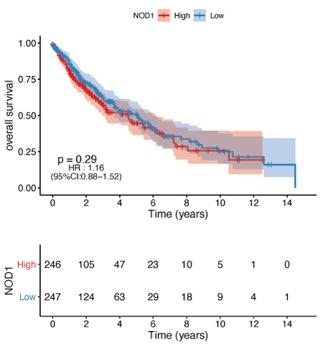

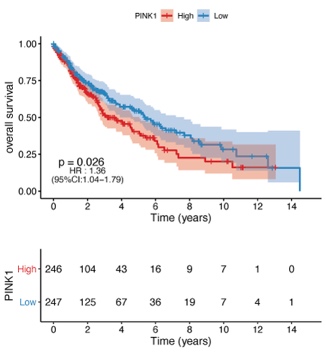

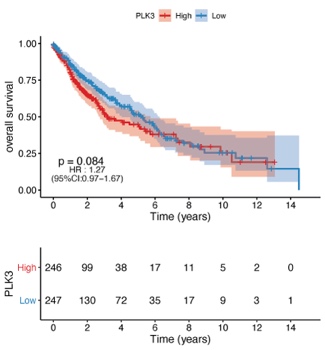
**
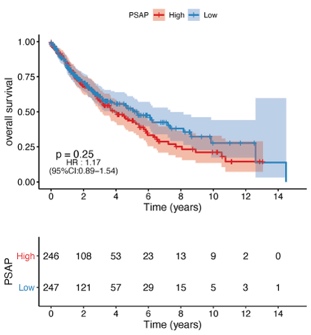


**
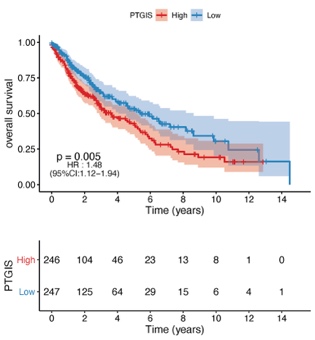

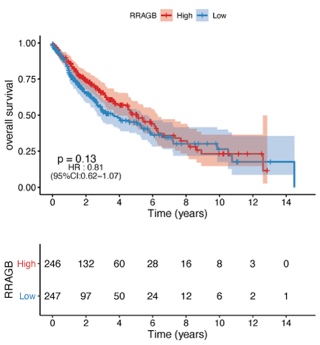

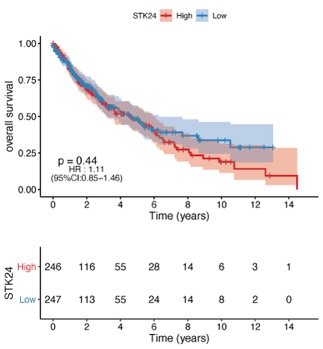

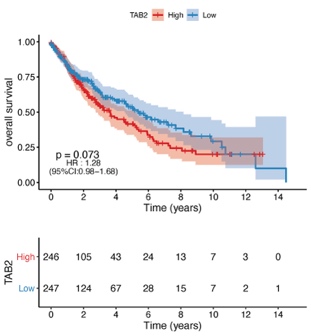
**

**
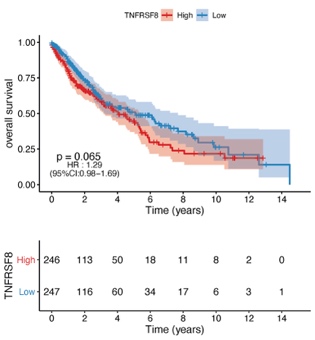

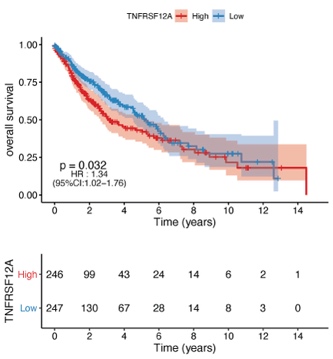

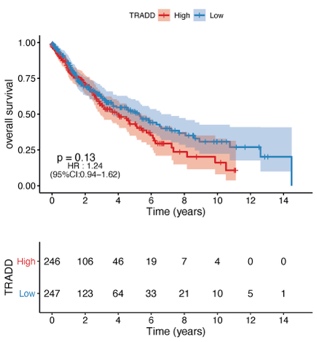

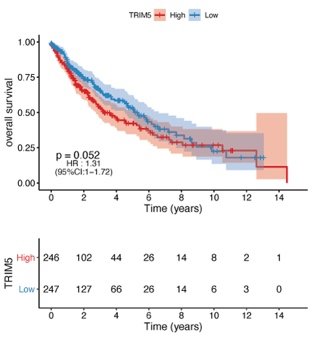
**

**Figure S2**. **Construction of cell death-associated prognostic signatures in the TCGA cohort.** (A) LASSO coefficient expression profiles. (B) The penalty parameter (λ) in the LASSO model was selected through 10-fold cross-validation.


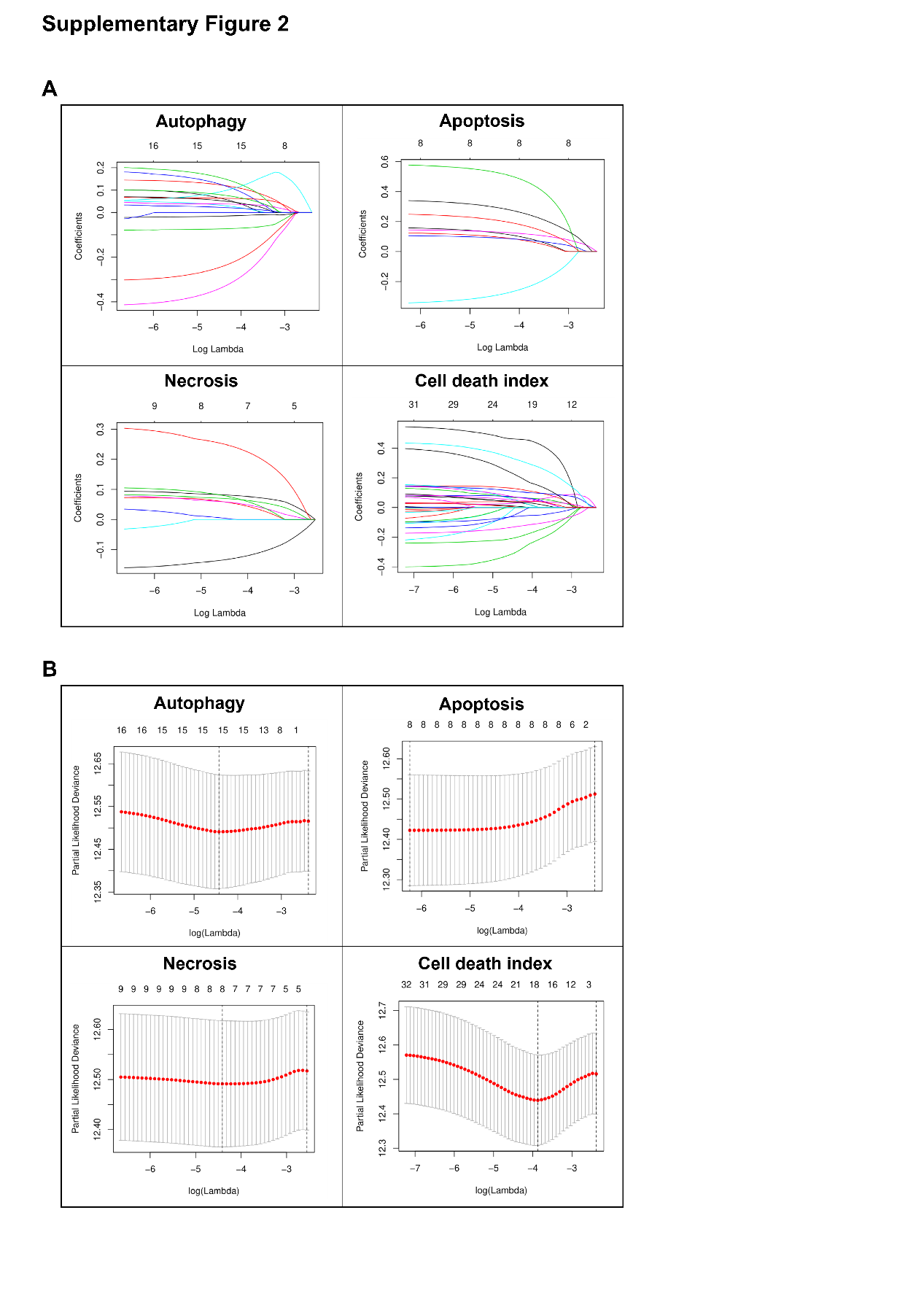

Supplement: Supplementary file 1 — Supplementary Material 1 [file 12931_2023_2402_MOESM1_ESM.docx]
